# Greenock Nursing Association

**Published:** 1921-03-12

**Authors:** 


### Greenock Nursing Association.

At Greenock, child welfare is sensibly included in the sphere of the district nurses, and the number of visits paid in this connection has trebled in the course of the year. In all departments the work shows a very marked growth. Unfortunately this means an increase of expenditure, and the accounts show a deficit of £167. With such good results behind them we feel confident the Committee will receive increased support to meet its obligations.

